# An improved protocol for bacteria identification by MALDI-TOF MS directly from positive blood cultures

**DOI:** 10.1007/s10096-023-04725-3

**Published:** 2023-12-19

**Authors:** Sara Cruz, David Abreu, Rosário Gomes, Inês Martins-Oliveira, Ana Silva-Dias, Blanca Perez-Viso, Rafael Cantón, Cidália Pina-Vaz

**Affiliations:** 1 Division of Microbiology, Department of Pathology, Faculty of Medicine, University of Porto, Porto, Portugal; 2 FASTinov SA, Porto, Porto, Portugal; 3 CINTESIS-Center for Health Technology and Services Research, Faculty of Medicine, University of Porto, Porto, Portugal; 4 Servicio de Microbiología, Hospital Universitario Ramón y Cajal and Instituto Ramón y Cajal de Investigación Sanitaria (IRYCIS), Madrid, Spain; 5 CIBER de Enfermedades Infecciosas (CIBERINFEC), Instituto de Salud Carlos III, 28029 Madrid, Spain

**Keywords:** MALDI-TOF, Mass spectrometry, Blood cultures, Rapid identification

## Abstract

FASTinov® developed a rapid antimicrobial susceptibility test that includes the purification of a bacterial suspension directly from positive blood cultures (BC). In order to streamline laboratory workflow, the use of the bacterial suspension obtained through FASTinov® sample *prep* was tested for identification (ID) by matrix absorption laser deionization–time of flight mass spectrometry (MALDI-TOF MS) (Bruker) in 364 positive BC, and its accuracy assessed comparing with the MALDI-TOF MS ID of the next-day subcultured colonies. FASTinov sample *prep *was highly reliable for rapid ID directly from BC with proportion of agreement of 94.9% for Gram-positive and 96.3% for Gram-negative bacteria.

## Introduction

Bloodstream infections are a serious health risk, being a significant cause of morbidity and mortality in the hospital [1]. The diagnosis relies on blood culture (BC) collection followed by the identification (ID) of the pathogen and subsequent antimicrobial susceptibility testing (AST). Due to their time-consuming nature, standard microbiology methods require approximately 2 days to produce an antibiogram from a positive blood culture.

The matrix-assisted laser desorption ionization–time of flight mass spectrometry (MALDI-TOF MS) method has revolutionized pathogen ID in clinical microbiology laboratories [2, 3]. Performed from colonies, ID is obtained 1 day earlier than standard methods but if performed directly from BC, the ID could be obtained 2 days earlier, avoiding subculturing with impact on patient outcome [4, 5]. Rapid ID of pathogens helps to provide an earlier transition from empiric antimicrobial therapy, which can fail in around 20% of cases [6]. However, with the increase in antimicrobial resistance, rapid ID alone is insufficient to decide the optimal antimicrobial therapy.

Several rapid susceptibility assays directly from BC have been recently described [7, 8]. However, as antimicrobial susceptibility is in certain cases species specific, there is a need to obtain a species-level ID of the pathogen before reporting the AST results. For instance, there are differences regarding some drugs and their breakpoint concentrations between *Pseudomonas* spp. and *Enterobacterales*, or even between different species of *Staphylococcus* such as *Staphylococcus aureus and Staphylococcus epidermidis* (EUCAST and CLSI protocols). For this reason, rapid ID methods are becoming essential in modern clinical microbiology laboratories.

The identification of microorganisms directly from a positive BC with MALDI-TOF MS can be performed using “in-house” protocols [9–15] or with commercial protocols. To our knowledge, five commercial protocols are available at present: the Sepsityper® kit (Bruker Daltonics) [16], the Vitek MS blood culture kit (bioMerieux, Inc.) [17], the rapid BACpro® II kit (Nittobo Medical Co.) [18], the FAST^TM^ system using the FAST-PBC Prep^TM^ cartridge (Qvella) [19], and the Accelerate Arc™ module with BC kit (Accelerate Diagnostics) [20, 21].

The FASTinov® Kit is an ultra-rapid AST directly performed from positive BC that provides results in a maximum of 2 h [8]. A proprietary sample *prep* is performed to obtain a purified bacterial suspension that is analyzed by flow cytometry. It includes a hemolytic agent that lyses red blood cells while maintaining the integrity of bacteria cells, which is an advantage that commercial kits such as the MBT Sepsityper® do not possess. Additionally, a density separating agent is used to remove the remaining blood cells, which results in a very clean bacterial suspension. A purified bacterial suspension is essential to assure the reliability of the results, as flow cytometry analysis is highly influenced by the presence of debris in the sample. This study aims to evaluate the possibility of using the FASTinov® sample *prep* from positive BC for rapid AST as a bacteria extraction protocol for MALDI-TOF MS identification, saving hands on time for both gram-positive and gram-negative bacteria.

## Methods

**Fig. 1:**
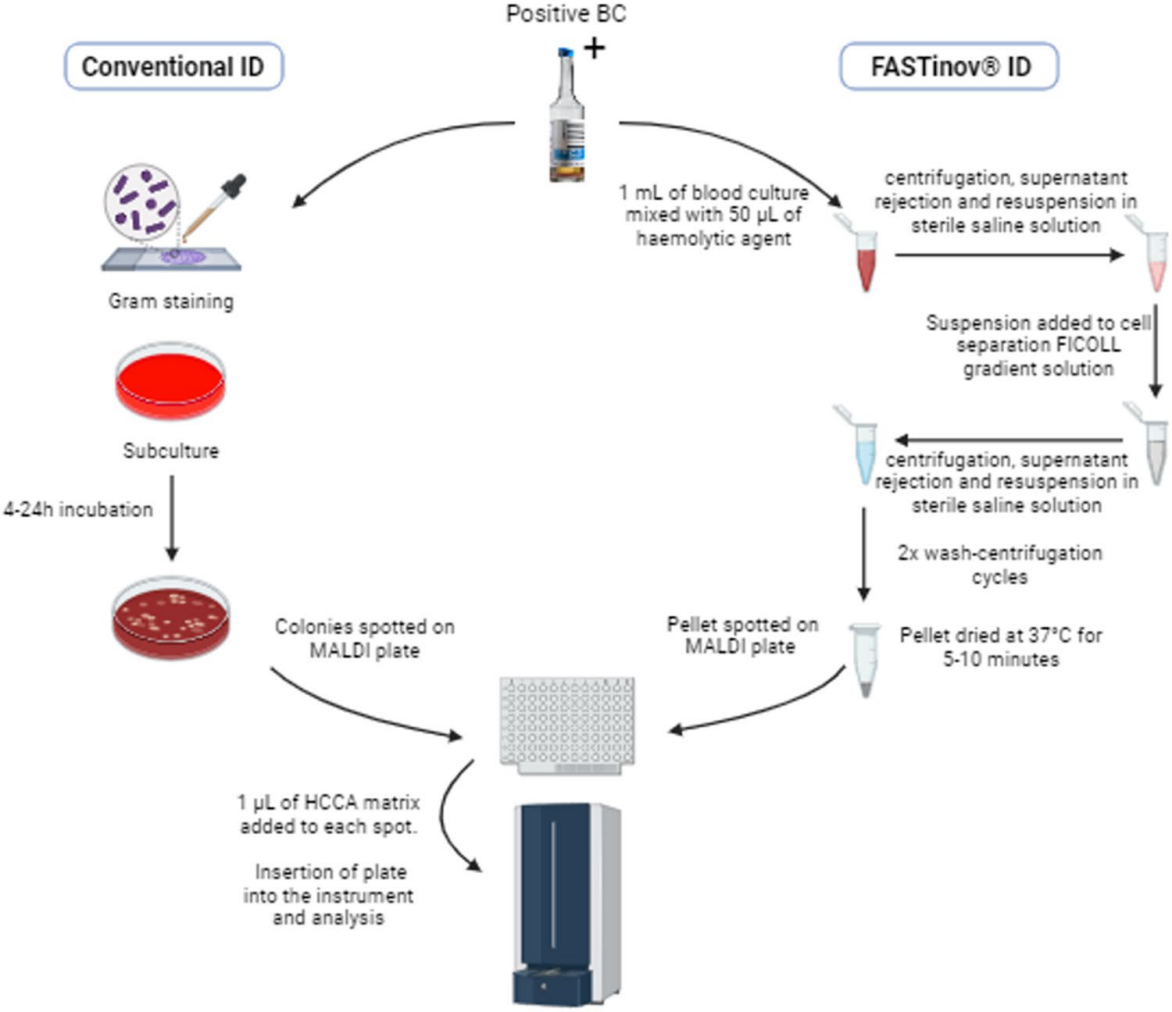
Flow chart of MALDI-TOF identification from a positive blood culture. The FASTinov® sample *prep* (right side of the figure) is compared with conventional identification of colonies by MALDI-TOF MS from a subculture (left side of the figure)

A total of 364 positive BC obtained from patients (BD BACTEC™) (177 gram-positive cocci, and 187 gram-negative bacilli) were processed according to the instructions for use of the FASTinov® AST kits and analyzed in a MALDI Biotyper sirius MALDI-TOF MS instrument from Bruker Daltonics (Hamburg, Germany), as shown in Fig. 1. The obtained results were compared to the ID obtained using subcultured bacterial colonies. To obtain a bacterial pellet from a positive BC, a sterile microcentrifuge tube was filled with 1 mL of the BC and mixed with 50 μL of the hemolytic agent included in the FASTinov kit, followed by vortex and centrifugation at 13,000 rpm for 1 min. The supernatant was discarded, and the pellet was resuspended in 1 mL of sterile saline solution; 500 μL of this suspension was gently transferred to the top of a microcentrifuge tube with 500 μL of a cell separation FICOLL gradient solution, and centrifugation was repeated for 1 min. The supernatant was discarded, and the pellet was washed with saline solution twice, and the pellet was dried at 37 °C for 5 min.

The obtained pellet was spotted directly on the MALDI-TOF MS target plate with a wooden toothpick in duplicate. One microliter of α-Cyano-4-hydroxycynnamic acid (HCCA) matrix was added to each spot and let dry before the insertion of the plate into the instrument. The target plate was placed in the Bruker MALDI Biotyper sirius and the analysis was initiated, using the Sepsityper sample-type option on the equipment. ID and respective scores obtained were recorded.

Score values were grouped in ranges defined according to the cut-off values on the MALDI Biotyper instrument. As reference method (scores obtained from next-day colonies), the standard sample-type option was chosen: scores ≤ 1.69 were considered to be unreliable (score group 1), scores 1.70–1.99 were considered to be low-confidence identifications (score group 2), and scores ≥ 2.00 were considered to be high-confidence identifications (score group 3) [22]. In order to analyze the samples extracted directly from positive blood cultures by the FASTinov® method, the *Sepsityper* sample-type option was chosen, and 3 groups of scores were defined. Scores ≤ 1.59 were considered to be unreliable (score group 1), scores 1.60–1.79 were considered to be low-confidence identifications (score group 2), and scores ≥ 1.80 were considered to be high-confidence identifications (score group 3) [23]. Only the highest score value of each sample was recorded.

## Results

**Fig. 2:**
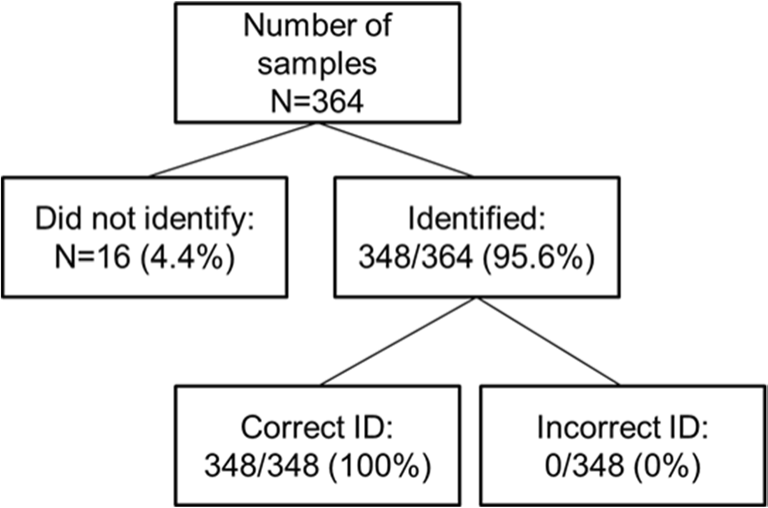
Flow chart of the number of blood culture samples tested on MALDI-TOF with the FASTinov sample *prep* as extraction protocol and its distribution between those that were identified or not; regarding those that were identified which ones were correctly identified (ID) or not

The global proportion of agreement (PA) of the FASTinov® ID method with the conventional reference technique (identification in subcultured colonies) was 95.6%. In 364 attempts, 16 (4.4%) failed to achieve identification, with no ID being provided; the strains that failed ID were 6 *Staphylococcus* spp., 3 *Enterococcus* spp., 1 *Escherichia coli*, 5 *Klebsiella* spp., and 1 *Pseudomonas aeruginosa* (Fig. 2). Detailed results were presented in Tables 1 and 2.

**Table 1:** Proportion of agreement (PA %) between the FASTinov® sample prep method for identification (ID) directly from positive blood cultures regarding gram-positive bacteria on MALDI-TOF MS: the ID produced by MALDI-TOF MS from colonies after subculture was the reference method

Gram-positive	*n*	PA %
*Staphylococcus spp.*	122	95.1
*S. aureus*	35	100
*S. capitis* *S. epidermidis*	4	75
	41	92.7
*S. haemolyticus*	6	83.3
*S. hominis*	35	97.1
*S. petrasii*	1	100
*Enterococcus spp.*	55	94.5
*E. faecalis*	39	92.3
*E. faecium*	15	100
*E. gallinarum*	1	100
Total	**177**	**94.9**

**Table 2:** Proportion of agreement (PA) between the FASTinov® sample prep method for identification (ID) directly from positive blood cultures on MALDI-TOF MS regarding gram-negative bacteria; the ID obtained from the colonies after subculture was the reference method

Gram-negative	*n*	PA %
*Escherichia coli*	83	98.8
*Klebsiella oxytoca*	3	66.7
*Klebsiella pneumoniae*	50	92.0
*Klebsiella variicola*	1	100
*Proteus mirabilis*	11	100
*Morganella morganii*	1	100
*Serratia marcences*	5	100
*Enterobacter cloacae*	5	100
*Pantoea agglomerans*	1	100
*Pseudomonas aeruginosa*	21	95.2
*Acinetobacter baumannii*	5	100
*Acinetobacter variabilis*	1	100
Total	**187**	**96.3**

The PA in gram-positive bacteria was 95.1% in the *Staphylococcus* spp. (116/122) and 94.5% in the *Enterococcus* spp. (52/55). Regarding gram-negative bacteria, the following values of PA were obtained: 98.8% in the *E. coli* (82/83), 90.7% in the *Klebsiella* spp. (49/54), and 95.2% in the *P. aeruginosa* (20/21). All samples for which an identification was obtained (*n* = 348) were in agreement with the reference method.

From those that were identified, scores above 1.8 were observed in 81.6% of gram-positive isolates; scores between 1.6 and 1.79 in 4.1%, and scores < 1.59 were observed in 14.3% of the cases. In gram-negatives, the scores were overall higher: 84.4% presented scores > 1.8, 15.6% presented score values 1.6–1.79, and none scoring < 1.59. The distributions per score group of the data obtained using standard method and FASTinov method are represented in Table 3.

**Table 3:** MALDI-TOF identification score results obtained after extracting bacteria directly from blood cultures (FASTinov sample prep). Results were distributed in score groups as previously discussed, and grouped with the reference method

		FASTinov sample *prep*			
		Score group 3 ≥ 1.80	Score group 2 1.60–1.79	Score group 1 ≤ 1.59	No ID
Reference method	Score group 3 ≥ 2.00	GP: 137 GN: 152			
	Score group 2 1.70–1.99		GP: 7 GN: 28		GN: 7
	Score group 1 ≤ 1.69			GP: 24	GP: 9

*ID*, identification; *GP*, gram-positive bacteria; *GN*, gram-negative bacteria

## Discussion

Rapid and accurate pathogen ID accompanied by a fast AST method in positive BC is essential for the management of septic patients. Rapid identification of blood pathogens allows the administration of effective antibiotics, which impacts on patient survival since it can improve clinical outcomes, as well as reduce hospitalization, length of stay, and hospital costs [23, 24]

The results obtained in this study show that the identification of gram-positive and gram-negative isolates using the FASTinov® ID method is highly reliable, with a global performance of 95.6% and no observed misidentifications. This could be due to the sample *prep* method itself, as the obtained bacterial suspension presents mainly intact bacteria and a reduced amount of debris. Other studies that have reviewed the performances of MALDI-TOF MS-based rapid ID from positive BC showed performances ranging from 60 to 99% at the species level [25]. It is difficult to directly compare these studies since the ID rates depend on the pretreatment method, the volume of the blood sample, the distribution of the microbial isolates, and the definition of the cut-off levels for species-level ID [26].

Although the misidentification rate varies between 0 and 4% in the literature [13, 17, 25], no discrepancy was found between the ID obtained through the FASTinov® method and the conventional method.

The global rate of unidentified isolates was 4.4% using the FASTinov sample *prep* method, which counts as an extraction protocol. Unidentified isolates using the FASTinov® ID method were slightly superior on Gram-positive bacteria, which are considered more difficult to identify by MS analysis due to their thick cell wall [13, 14]. The *Staphylococcus* spp. isolates unidentified by the FASTinov® ID method were all coagulase-negative *Staphylococci*, which was similar to other studies [13]. Formic acid was not used prior to the addition of the matrix, which could have further improved the identification rate.

The rate of unidentified isolates has been reported to be between 10 and 13% in studies without an extraction protocol [9, 27]. In Barnini et al., where only an extraction protocol was used in Gram-positive bacteria, the rate of unidentified isolates was reported to be 6% [13]. In a study with a protocol used only to ID *Enterobacterales* and *Pseudomonas aeruginosa,* all the isolates were identified from the pellet [10]. The FAST-PBC Prep^TM^ cartridge (Qvella) is an automated system with an extraction protocol for both gram-positive and gram-negative bacteria and showed correct ID in 94% or 90% of samples, respectively. More recently, a similar equipment, the Accelerate Arc™ module has been described [21]. Both systems have the advantage of being automated but take more than 40 min to provide results and are expensive. The FASTinov sample *prep*, included in the FASTinov rapid AST kit, allows the rapid and accurate ID from a positive BC without additional costs. After performing ID using the FASTinov method, the lab has the ability to better decide which workflow the positive BC should take: either a conventional AST or rapid AST. For labs that are already using FASTinov® AST, the possibility of performing ID using the same procedure is a meaningful advantage, as the 2-in-1 FASTinov® sample *prep* optimizes the laboratory workflow, reducing the hands on time of the lab technicians. Other sample *prep* systems also provide accurate results and thus could be viable alternatives to FASTinov sample *prep*; however, the 2-in-1 aspect of FASTinov would be lost. Despite its many advantages, the FASTinov sample *prep* is not readily available as an isolated kit and still requires manual steps.

In conclusion, using the FASTinov® method as a bacteria extraction protocol from positive BC for ID of gram-positive and gram-negative isolates is highly reliable. FASTinov® ultra-rapid AST sample *prep* can be used to run in parallel both ID with MALDI-TOF MS and ultra-rapid AST. This combination leads to the saving of hands-on time while providing excellent accuracy for both gram-positive and gram-negative bacteria. Using the FASTinov® sample preparation method, we are able to provide ID and AST directly from positive BC with 3–5 min of hands-on time, and a maximum of 2 h.

This study was not performed on Vitek® MS from bioMérieux, which is a limitation, but we do not expect very different results as other methods have been presenting similar outcomes.

## Data Availability

All data supporting the study is not publicly available but can be accessed upon reasonable request to the corresponding author.
